# Swimming Training Modulates Nitric Oxide-Glutamate Interaction in the Rostral Ventrolateral Medulla in Normotensive Conscious Rats

**DOI:** 10.3389/fphys.2016.00221

**Published:** 2016-06-13

**Authors:** Hiviny de A. Raquel, Gustavo S. Masson, Barbara Falquetto Barna, Nágela G. Zanluqui, Phileno Pinge-Filho, Lisete C. Michelini, Marli C. Martins-Pinge

**Affiliations:** ^1^Department of Physiological Sciences, Center of Biological Sciences, State University of LondrinaLondrina, Brazil; ^2^Department of Physiology & Biophysics, Institute of Biomedical Sciences, University of São PauloSão Paulo, Brazil; ^3^Department of Pathological Sciences, Center of Biological Sciences, State University of LondrinaLondrina, Brazil

**Keywords:** baroreflex, RVLM, nitric oxide synthase, heart rate, arterial pressure

## Abstract

We evaluated the effects of swimming training on nitric oxide (NO) modulation to glutamate microinjection within the rostral ventrolateral medulla (RVLM) in conscious freely moving rats. Male Wistar rats were submitted to exercise training (Tr) by swimming or kept sedentary (Sed) for 4 weeks. After the last training session, RVLM guide cannulas and arterial/venous catheters were chronically implanted. Arterial pressure (AP), heart rate (HR), and baroreflex control of HR (loading/unloading of baroreceptors) were recorded in conscious rats at rest. Pressor response to L-glutamate in the RVLM was compared before and after blockade of local nitric oxide (NO) production. In other Tr and Sed groups, brain was harvested for gene (qRT-PCR) and protein (immunohistochemistry) expression of NO synthase (NOS) isoforms and measurement of NO content (nitrite assay) within the RVLM. Trained rats exhibited resting bradycardia (average reduction of 9%), increased baroreflex gain (Tr: −4.41 ± 0.5 vs. Sed: −2.42 ± 0.31 b/min/mmHg), and unchanged resting MAP. The pressor response to glutamate was smaller in the Tr group (32 ± 4 vs. 53 ± 2 mmHg, *p* < 0.05); this difference disappeared after RVLM pretreatment with carboxy-PTIO (NO scavenger), Nw-Propyl-L-Arginine and L-NAME (NOS inhibitors). eNOS immunoreactivity observed mainly in RVLM capillaries was higher in Tr, but eNOS gene expression was reduced. nNOS gene and protein expression was slightly reduced (−29 and −9%, respectively, *P* > 0.05). Also, RVLM NO levels were significantly reduced in Tr (−63% vs. Sed). After microinjection of a NO-donor, the attenuated pressor response of L-glutamate in Tr group was restored. Data indicate that swimming training by decreasing RVLM NO availability and glutamatergic neurotransmission to locally administered glutamate may contribute to decreased sympathetic activity in trained subjects.

## Introduction

Aerobic training induces adaptations in central autonomic areas involved in the control of the cardiovascular system (Ichiyama et al., 2002; Martins-Pinge, 2011). Such changes modify the parasympathetic and sympathetic outflow to heart and vessels, with robust changes in the peripheral sympathetic activity (Mitchell and Victor, 1996). In this sense, the rostral ventrolateral medulla (RVLM), the main sympathetic output for heart and blood vessels (Dampney, 1994), emerges as a potent target for modulatory action of exercise on autonomic control. However, only few studies have evaluated functional RVLM changes in animals previously submitted to exercise training (Becker et al., 2005; Martins-Pinge et al., 2005; Mueller, 2007; Ogihara et al., 2014).

RVLM premotor neurons driving the excitatory tone to the sympathetic preganglionic neurons are mainly glutamatergic (Guertzenstein and Silver, 1974; Dampney, 1994). Indeed microinjections of L-glutamate in the RVLM produce pressor response in both anesthetized (Willette et al., 1987) and awake rats (Bachelard et al., 1990; Martins-Pinge et al., 2005). In addition, studies have shown increased release of glutamate within the RVLM during static muscle contractions (Caringi et al., 1998; Lillaney et al., 1999; Ishide et al., 2003), suggesting this neurotransmitter is directly involved in the exercise pressor reflex (Ally, 1998).

Interestingly, we previously observed smaller pressor responses to RVLM glutamate administration in conscious rats previously submitted to a protocol of swimming training (Martins-Pinge et al., 2005). Experimental evidence indicated that exercise, through flow-induced shear stress, increases nitric oxide (NO) production to cause local vasodilation (Green et al., 2004; McAllister and Laughlin, 2006). It was also demonstrated the presence of NO synthase (NOS) isoforms in the RVLM (Chan et al., 2001), which, under physiological conditions are able to activate the local synthesis of NO. It has been proposed that NO is produced during the activation of NMDA receptors, suggesting its involvement in the activation of glutamatergic pathways (Wu et al., 2001). Indeed the involvement of NO in glutamatergic neurotransmission within the RVLM was previously reported (Martins-Pinge et al., 1999). However, the production of NO in the RVLM as well as its potential effects on the autonomic control of the circulation following exercise training has not been investigated yet.

Knowing that RVLM neurons express NOS isoforms and that NO has a functional role in glutamatergic neurons (Dampney, 1994), we hypothesized that exercise would change RVLM NO availability, thus contributing to the small pressor response observed in swimming-trained rats. Therefore, in the present study we analyzed the pressor response to glutamate microinjection in the RVLM in sedentary and swimming-trained rats before and after pretreatment with NOS blockers and NO scavenger within the RVLM. In addition, we compared gene and protein expression of eNOS and nNOS as well as NO availability in the RVLM of sedentary and trained rats. We also performed L-glutamate microinjections in RVLM of sedentary and trained rats previously treated with a NO-donor.

## Materials and methods

### Animals

Adult male Wistar rats, weighing 220–240 g at the beginning of protocols were used. They were housed at the Central Animal Facility of the State University of Londrina, Brazil at controlled room temperature (22 ± 1°C) with a 12-h dark-light cycle and free access to standard chow and water. All surgical and experimental protocols were in accordance and recommendations of Brazilian National Council for Animal Experimentation Control (CONCEA) and approved by the Ethics Committe of the State University of Londrina, Brazil (process number: 35247.2011.45).

### Exercise training protocol

The animals were randomly allocated to two groups: trained group (Tr) submitted to swimming training and sedentary group (Sed), which was not submitted to the exercise protocol. The swimming training, according to Martins-Pinge et al. (2005), was conducted between 11:00 AM and 1:00 PM in a glass tank (4000 cm^2^ of surface area, 60 cm deep) with water heated to 31 ± 1°C. The training protocol consisted of 4 weeks of swimming being carried out 60 min per day, 5 times a week. During the first week the animals swam 15 min on the 1st day, 30 min the 2nd day, 45 min on the 3rd day and 60 min from the 4th day on.

### Guide cannula implantation in the RVLM

One day after the last exercise session the rats were anesthetized with sodium pentobarbital (40 mg/kg, *ip*) and underwent stereotaxic surgery for implantation of guide cannulas directed to RVLM. Rats were placed in the stereotaxic apparatus (David Kopf) with the incisor bar 5 mm below the interaural line, according to Martins-Pinge et al. (1997). At the end of this procedure, the animals received a prophylactic dose of antibiotic (40.000 IU) and returned to the Animal Facility for 3 days for surgical recovery.

### Artery and vein catheterization and cardiovascular recordings

Twenty-four hours before the experiments, the rats were again anesthetized (tribromoethanol, 250 mg/kg, *ip*) for implantation of catheters in the femoral artery and vein for arterial pressure (AP) and heart rate (HR) recordings and drugs administration, respectively. The arterial cannula was attached to a pressure transducer (Model MLT0380, Powerlab) connected to a computerized system (Powerlab, AD Instruments) and ~30 min were allowed for adaptation to the environment (individual cage in a quiet room). Baseline AP and HR were continuously recorded in conscious freely-moving rats for 30 min.

### Baroreflex testing

Baroreflex function was analyzed by loading/unloading of baroreceptors with intravenous bolus injections (100 μL) of phenylephrine (0.1 up to 12.8 μg/kg) and sodium nitroprusside (0.2 up to 25.6 μg/kg). Subsequent injections were not made until the returning of MAP and HR to basal values. MAP and HR values were measured immediately before (control) and at the peak of each response. Baroreceptor reflex control of HR, determined for each rat, was estimated by the sigmoidal logistic equation fitted to data points, as described previously (Kent et al., 1972; Head and Mccarty, 1987). The equation linking HR responses to pressure changes was: HR = P1+P2/[1+eP3(BP–P4)], where P1 = lower HR plateau, P2 = HR range, P3 = the curvature coefficient and P4 = BP50 (the value of MAP at half of the HR range). The average gain of baroreflex function (BrS) was also calculated (Kent et al., 1972; Head and Mccarty, 1987). Baroreflex testing was performed in groups of trained and sedentary rats without guide cannula implantation.

### RVLM microinjections

The protocol consisted of initial unilateral microinjection of L-glutamate (5 nmol/100 nL). After microinjection, MAP and HR responses were followed for 5–10 min recovery interval for returning of cardiovascular parameters to baseline values. Then, RVLM was treated with one of the following drugs: saline 0.9% (Sed: *n* = 4; Tr: *n* = 5) or NO scavenger, Carboxy-PTIO (1 nmol/100 nL) (Sed: *n* = 8; Tr: *n* = 10) or the nNOS inhibitor Nw-Propyl-L-Arginine (4 nmol/100 nL) (Sed: *n* = 8; Tr: *n* = 10) or the unspecific NOS inhibitor L-NAME (15 nm/100 nL) (Sed: *n* = 8; Tr: *n* = 7). RVLM L-glutamate (5 nmol/100 nL) microinjection was then repeated and MAP and HR responses were followed for 5–10 min up to the return of cardiovascular parameters to baseline values.

Another protocol consisted of previous treatment of RVLM with DeaNonoate (an NO-donor, 50 nmol/100 nL) followed by microinjection of L-glutamate in the RVLM of sedentary and trained animals (Sed: *n* = 4; Tr: *n* = 4).

The concentrations of drug used were based on the following literatures: L-glutamate, and L-NAME:. Martins-Pinge et al. (2007); carboxy-PTIO and N-Propyl-L-arginine: Busnardo et al. (2010). Dea-NONOate: Yao et al. (2007). At the end of each experimental protocol, the animals were euthanized with an extra dose of anesthetic and then held marking procedures of microinjection sites and removal of the brain for subsequent histological analysis.

### Confirmation of RVLM microinjections

At the end of the experimental protocols, the rats were euthanized with an overdose of sodium pentobarbital. Sites of RVLM administrations were marked by microinjection of Evans blue dye (2%/100 nL). Brain was removed and stored in 10% formaldehyde for subsequent histological analysis. Sequential slices (40 μm) of brainstem were cut in a cryostat, placed in gelatinized slides and stained with 1% neutral red. The sections were examined microscopically with the aid of a rat brain atlas (Paxinos, 1998). Only rats with confirmed RVLM microinjection were included in experimental groups (see **Figure 2F**).

### Tissue harvesting for qPCR and immunohistochemistry assays

Gene and protein expression in the RVLM were analyzed in other groups of sedentary and trained rats not submitted to RVLM cannulation. At the end of experimental protocols, rats were deeply anesthetized (60 mg/kg pentobarbital *i.p*) and the brains perfused with phosphate-buffered saline (PBS 0.1 M, pH 7.4, ~30 mL/min for 4–5 min, via a left ventricle cannula) immediately after the respiratory arrest (Cavalleri et al., 2011). In 8–10 rats/group, fresh brains were rapidly removed and frozen in a dry ice box. Bilateral punches of RVLM were obtained from frozen brain stem sections (rostral to the Obex, 1000–1200 μm of thickness) and stored in a deep freezer in individual eppendorfs with 1 mL Trizol^®^ until processing. In the remaining 3–4 rats/group, after the initial perfusion with PBS, brains were fixed with 4% paraformaldehyde (PFA, 30 mL/min for 20–30 min). Brains were removed from the skull, post-fixed in 4% PFA for 24–48 h. Series of coronal sections (40 μm) from brain stem were cut using the Leica-CM3050 cryostat and stored in a cryoprotectant solution (20% glycerol plus 30% ethylene glycol in 50 mM phosphate buffer, pH 7.4, −20°C) for up to 2 weeks until histological processing (Schreihofer and Guyenet, 1997).

### Real-time qPCR

RVLM mRNA expression was estimated in Tr and Sed by the real-time qPCR. The total RNA was extracted using the Trizol^®^, dissolved in 10 μL of DEPC water and stored at -80°C. Then, the samples were treated with DNAse I for cDNA synthesis by reverse transcription (Revert Aid TMM-MuLV Reverse Transcriptase) according to the protocol supplied by the manufacturer. The cDNA obtained was then stored at −20°C. The samples were subjected to amplification by Real Time qPCR method using Platinum SYBRGreen qPCR Supermix-UDG (Cavalleri et al., 2011) and specific primers for the two NOS isoforms: eNOS (Gene Bank: NM_021838.2/Fragment Size: 94pb, sense primer: GCCAAACAGGCCTGGCGCAA, antisense primer: GTGCTGTCCTGCAGTCCCGA) and nNOS (Gene Bank: NM_0522799.1/Fragment Size: 118pb, sense primer: CGCTACGCGGGCTACAAGCA, antisense primer: GCACGTCGAAGCGGCCTCTT). mRNA expression was estimated by semi-quantitative real time PCR (7500 Real-Time PCR System). The specificity of the SYBRGreen assays was confirmed by analysis of the melting points of the curves. The endogenous gene was the hypoxanthine guanine phosphoribosyl transferase—HPRT (Gene bank: NM_012583.2/Fragment Size: 125pb), which is continuously expressed in all cells of the body and not altered by physical training (Cavalleri et al., 2011). Analysis of gene expression was made by the geNorm software VBA applet for Microsoft Excel, considering the values of threshold cycle (Ct) and the ΔΔCt method (Cavalleri et al., 2011). The results were expressed as fold increase. All reagents and primers were purchased from Invitrogen (San Diego, CA, USA).

### Immunohistochemistry

Endothelial nitric oxide syntase (eNOS) and neuronal nitric oxide syntase (nNOS) immunoreactivities were detected in sequential brain stem slices using mouse anti-eNOS/NOS Type III antibody (1:200, BD Transduction Laboratories) and mouse anti-nNOS (1:200, BD Transduction Laboratories), respectively, as previously described (Llewellyn-Smith et al., 2005; Barna et al., 2012). Biotin-SP-conjugated AffiniPure Donkey Anti-Mouse IgG (H+L) (1:500, Jackson Immuno Research Laboratories—immunoperoxidase assay) was used as the secondary antibody. Brain stem slices were mounted in sequential rostrocaudal order; slides were dried and covered with Krystalon (EMD Chemicals Inc, NJ). Brain sections were analyzed in a Zeiss Axioskop 2 microscope (Oberkochen, Germany) to check the location of neurons and vessels marked. RVLM neurons immunoreactive to nNOS and vessels immunoreactive to eNOS were identified and quantified by a blind investigator. The images from both experimental groups were digitized with identical acquisition settings. Image analysis was performed with Image J software (Wright Cell Imaging Facility—Toronto Western Research Institute, ON). An automated tracing procedure that incorporated the threshold paradigm was applied the acquired images. The background intensity was calculated from random adjacent areas in the RVLM. ROIs of predetermined sizes were used to determine the density of eNOS threshold signal. Values for each area per rat were averaged to obtain the mean value for each experimental group.

### Nitrite levels in RVLM

Indirect NO concentration in RVLM was estimated in punches obtained from sedentary (*n* = 10) and trained (*n* = 8) rats through the measurement of nitrite as described previously (Navarro-Gonzálvez et al., 1998; Panis et al., 2011). Group Samples (Sed: 3.6 ± 0.3 and Tr: 3.5 ± 0.4 mg), keeping the concentration of 100 mg of wet weight tissue per milliliter of PBS, were used. All reagents for the nitrite assay were obtained from Sigma Chemical Co. The results were expressed in uM of nitrite/mg of RVLM tissue.

### Statistical analysis

All data are reported as mean ± SEM. Nitrite concentration, gene and protein expression in Gtr and Gsed, baroreflex sensitivity and MAP and HR responses determined by RVLM drugs microinjections in both groups were compared by *Student T test* or *paired “T” test* as appropriate. Differences between groups were analyzed by one-way ANOVA followed by Newman-Keuls as the *post hoc* test. Differences were considered significant when P < 0.05.

## Results

**Figure 1:**
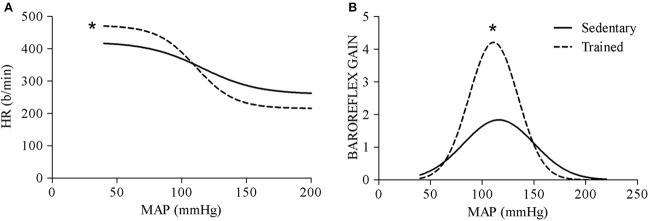
**(A)** Sigmoidal function curves expressing the relationship between heart rate (HR) and mean arterial pressure (MAP) during loading and unloading of baroreceptors in conscious sedentary and swimming-trained rats. **(B)** Changes of baroreflex gain at different pressure levels. The parameters of curves are: Sedentary—Lower plateau = 259 b/min, HR range = 162 b/min, BP50 = 116 mmHg, Maximal gain sensitivity = 0.047 b/min/mmHg; Trained—Lower plateau = 215 b/min, HR range = 257 b/min, BP50 = 111 mmHg, Maximal gain sensitivity = 0.067 b/min/mmHg. Significance (*p* < 0.05): ^*^ vs. sedentary group.

In all groups of rats studied, swimming training was accompanied by resting bradycardia (average reduction of ~9%, when compared to respective sedentary groups, Table 1). In addition we observed improved baroreceptor reflex control of HR (Figure 1A) and increased baroreflex gain (Figure 1B) in the trained animals compared to sedentary controls (Gtr: −4.41 ± 0.5 vs. Gsed: −2.42±0.31 b/min/mmHg, *P* < 0.05). These responses confirmed the efficacy of exercise training to improve cardiovascular control. Also, as observed in Table 1, swimming training did not change baseline MAP in the normotensive groups of rats.

**Table 1:** **Resting values of mean arterial pressure (MAP) and heart rate (HR) and basal values of MAP before the first and the second Glutamate microinjections in sedentary and swimming trained groups treated with Saline, Carboxi-PTIO, Nw-Propyl-L-Arginine, and L-NAME within the RVLM**.

	**Saline**		**Carboxi-PTIO**		**Nw-Propyl-L-Arginine**		**L-NAME**	
	**Sedentary**	**Trained**	**Sedentary**	**Trained**	**Sedentary**	**Trained**	**Sedentary**	**Trained**
**RESTING VALUES**								
MAP (mmHg)	107 ± 2 (*n* = 4)	110 ± 2 (n = 5)	112 ± 2 (*n* = 8)	112 ± 4 (*n* = 10)	112 ± 3 (*n* = 8)	111 ± 2 (*n* = 10)	112 ± 1 (*n* = 8)	109 ± 1 (*n* = 9)
HR (b/min)	356 ± 1 (*n* = 4)	315 ± 12^*^ (*n* = 5)	363 ± 4 (*n* = 8)	336 ± 6 ^*^ (*n* = 10)	363 ± 9 (*n* = 8)	338 ± 4 ^*^ (*n* = 10)	377 ± 5 (*n* = 8)	348 ± 6^*^ (*n* = 9)
**BASAL MAP DURING RVLM INJECTIONS**								
Before treatment	118 ± 6 (*n* = 4)	124 ± 11 (*n* = 5)	126 ± 2 (*n* = 8)	125 ± 2 (*n* = 10)	129 ± 3 (*n* = 8)	123 ± 1 (*n* = 10)	119 ± 2 (*n* = 8)	116 ± 2 (*n* = 9)
After treatment	127 ± 4 (*n* = 4)	120 ± 4 (*n* = 5)	124 ± 2 (*n* = 8)	125 ± 2 (*n* = 10)	130 ± 3 (*n* = 8)	128 ± 2 (*n* = 10)	121 ± 3 (*n* = 8)	116 ± 3 (*n* = 9)

* *(p ≤ 0.05 compare to corresponding sedentary group; Student t-test)*.

**Figure 2:**
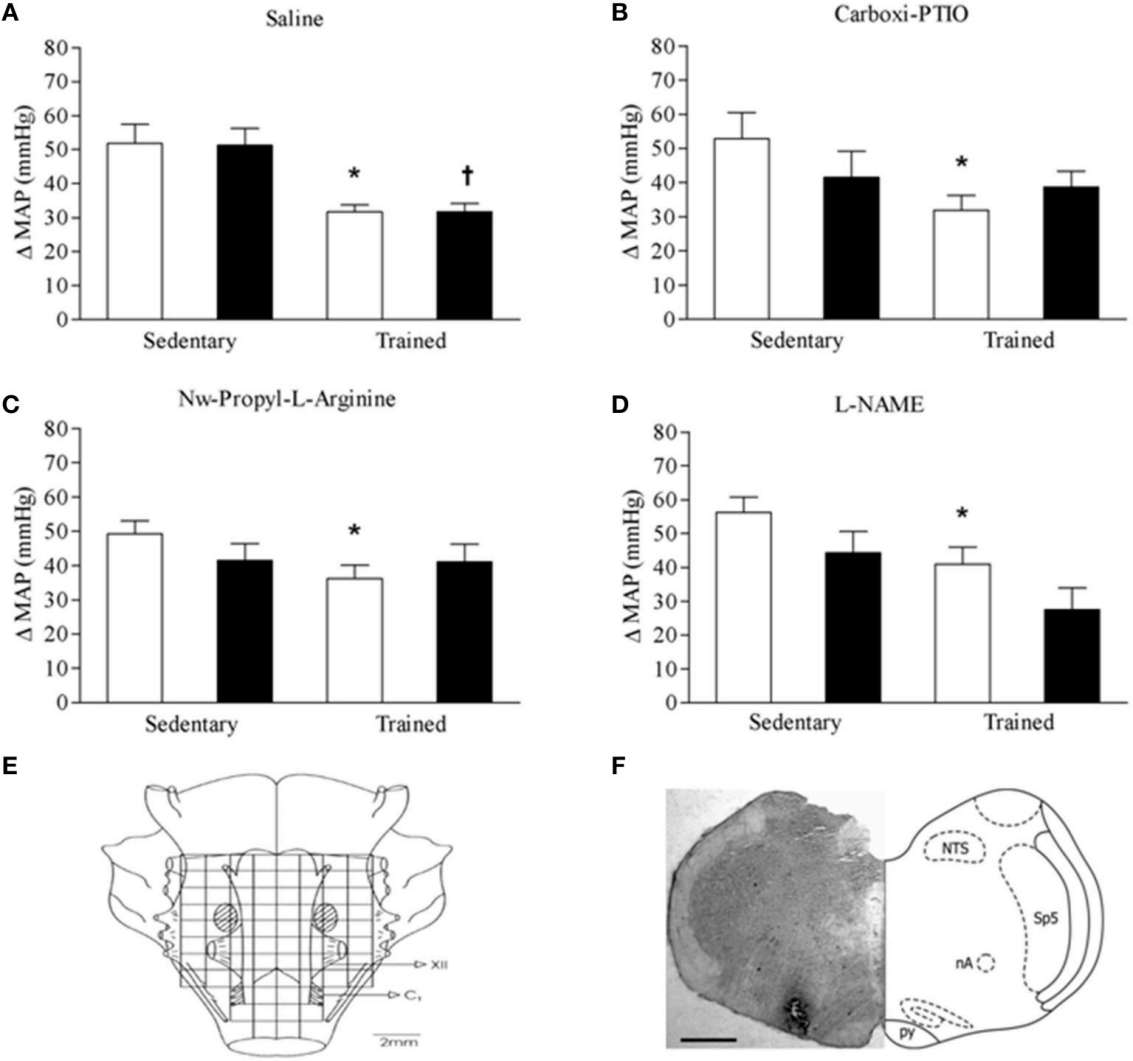
**Mean Arterial Pressure (MAP) responses (ΔMAP) to unilateral L-glutamate microinjection in the RVLM of sedentary and trained rats before (open bars) and after (filled bars) pretreatment of RVLM with Saline (A), Carboxy-PTIO (B), n-Propyl-L-Arginine (C), and L-NAME (D)**. Significances (*p* < 0.05) are vs. sedentary group: ^*^ before; ^†^ after RVLM treatment. Scheme on panel **(E)** and photomicrograph on **(F)** illustrate the localization of microinjections within the RVLM area. XII cranial nerve; C1, first cervical nerve; NTS, nucleus of the solitary tract; nA, nucleus ambiguous; Sp5, spinal trigeminal nucleus; py, pyramidal tract; RVLM, rostral ventrolateral medulla. Scale bar in **(F)** represents 500 μm.

Accordingly with previous data (Martins-Pinge et al., 2005), the L-glutamate administrations in RVLM elicited marked pressure increases that were significantly reduced by swimming training, and the same was observed in all groups analyzed here before the different treatments (Figure 2). In sedentary rats, the pressor response to L-Glu in the RVLM before and after local microinjections of saline were, respectively, 53 ± 2 mmHg (Figure 2A, open bar) and 52 ± 6 mmHg (Figure 2A, dark bar) and, in the trained rats were 35 ± 2 mmHg (Figure 2A, open bar) and 32 ± 2 mmHg (Figure 2A, dark bar). However, previous microinjection of Carboxi-PTIO canceled the differences in the pressor responses to L-glutamate between sedentary and trained animals (Figure 2B). The RVLM previous treatment with Nw-Propyl-L-Arginine, a nNOS inhibitor, caused the same results (Figure 2C). A similar observation was made after L-NAME (a nonselective NOS inhibitor) administration in the RVLM, in which, the pressor responses to L-Glu in the RVLM were not different between sedentary and trained rats (Figure 2D).

As depicted in the map of the ventral surface of the medulla (Figure 2E) and in a coronal section of the brain stem (Figure 2F), dye injection at the end of experiments confirmed that all microinjections were directed to the RVLM.

**Figure 3:**
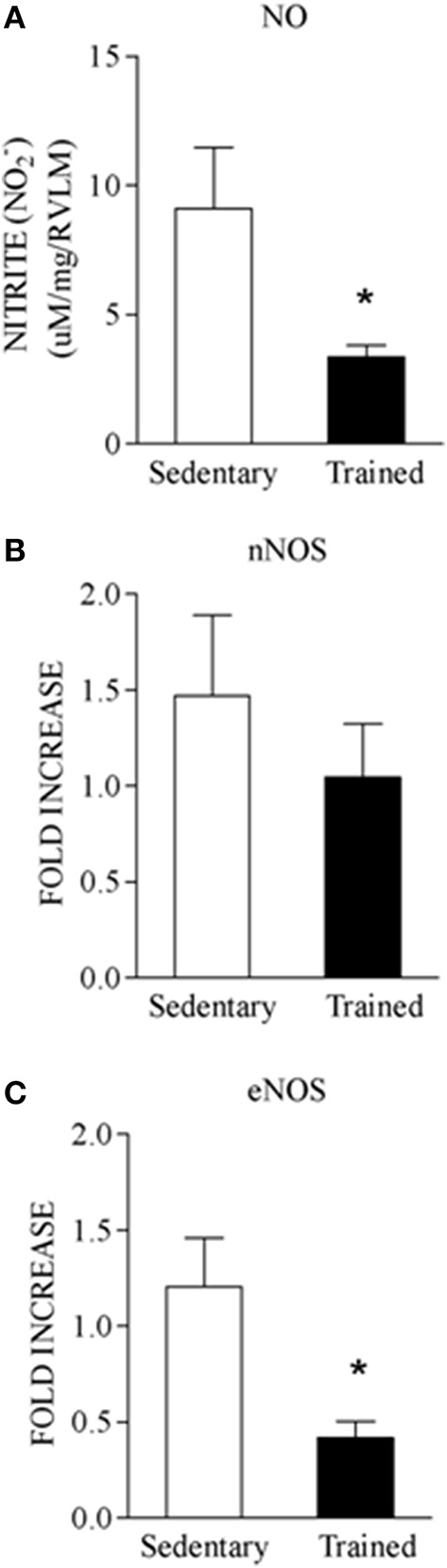
**Comparison of nitrite concentration (A), mRNA gene expression of nNOS (B), and eNOS (C) in punches of RVLM of sedentary and trained rates**. Significance (^*^p < 0.05) vs. sedentary group.

**Figure 4:**
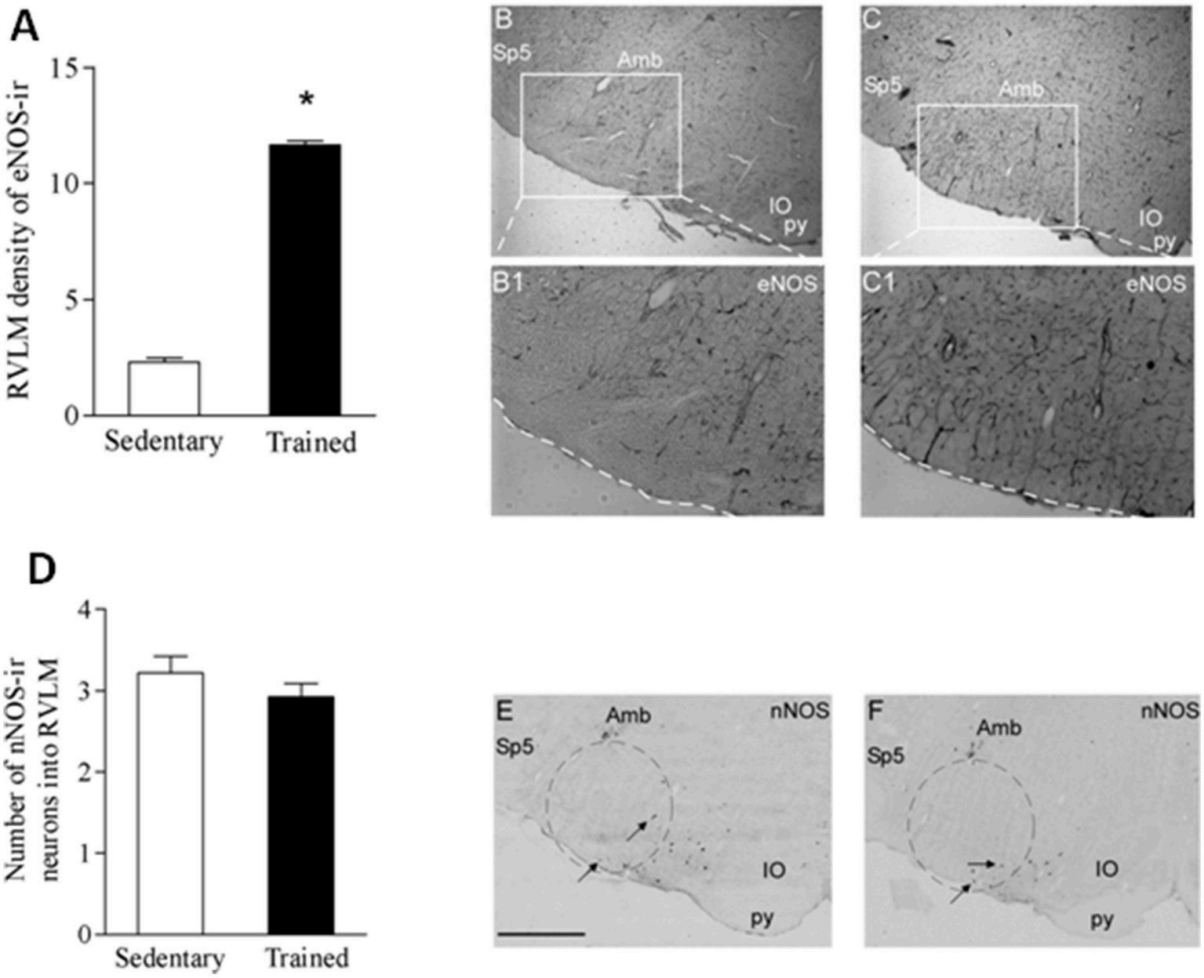
**Immunohistochemistry (ir) for eNOS (upper panels) and nNOS (lower panels) in the RVLM of sedentary (Gsed) and trained (Gtr) groups. (A)** Quantification of RVLM eNOS density in Gsed and Gtr. Significance (*p* < 0.05) ^*^ vs. sedentary group. **(B,C)** Representative photomicrographs of RVLM eNOS-ir in 2 different magnifications in sedentary **(B,B1)** and trained rats **(C,C1)**. **(D)** Quantification of nNOS-ir/RVLM slice in Gsed and Gtr. **(E,F)** Representative photomicrographs showing RVLM nNOS-positive neurons in sedentary **(E)** and trained **(F)** rats. Scale bar represents 200 μm. Sp5, spinal trigeminal nucleus; py, pyramidal tract. Black arrows indicate NOS-positive neurons.

The gene expression of NOS isoforms within the RVLM was also analyzed in both groups. There was a marked reduction of RVLM eNOS expression in the trained group (from 1.20 ± 0.25 in Sed to 0.41 ± 0.08 in Tr, *P* < 0.05, Figure 3C) and a small decrease in nNOS mRNA expression that did not attain significance (Sed: 1.46 ± 0.42 vs. Tr: 1.04 ± 0.27, *P* > 0.05, Figure 3B). Immunohistochemistry for eNOS in the RVLM revealed a “blood vessels pattern,” confirming the presence of eNOS mainly in the endothelium of capillaries within the RVLM (Figures 4B,B1,C,C1). In agreement with the increased capillary supply observed in brain areas of trained animals (Dunn et al., 2012; Huang et al., 2013) quantitative analysis showed that trained rats exhibited increased eNOS immunoreactivity when compared to sedentary controls (Sed: 2.3 ± 0.2 vs. Tr: 11.7 ± 0.2, *P* < 0.05, Figure 4A). On the other hand, nNOS immunoreactivity in the RVLM was present essentially in neuronal cell bodies (Figures 4E,F). There was a slight training-induced reduction in RVLM nNOS positive neurons (-9.4%), but values did not attain significance (Sed: 3.2 ± 0.2 vs. Tr: 2.9 ± 0.1, *P* > 0.05, Figure 4D). Interestingly, the comparison of nitrite concentration indicated lower NO availability within the RVLM of trained rats (Sed: 9.1 ± 2.0 vs. Tr: 3.4 ± 0.4 μM of nitrite/mg of tissue, a reduction of 63%, *P* < 0.05, Figure 3A).

**Figure 5:**
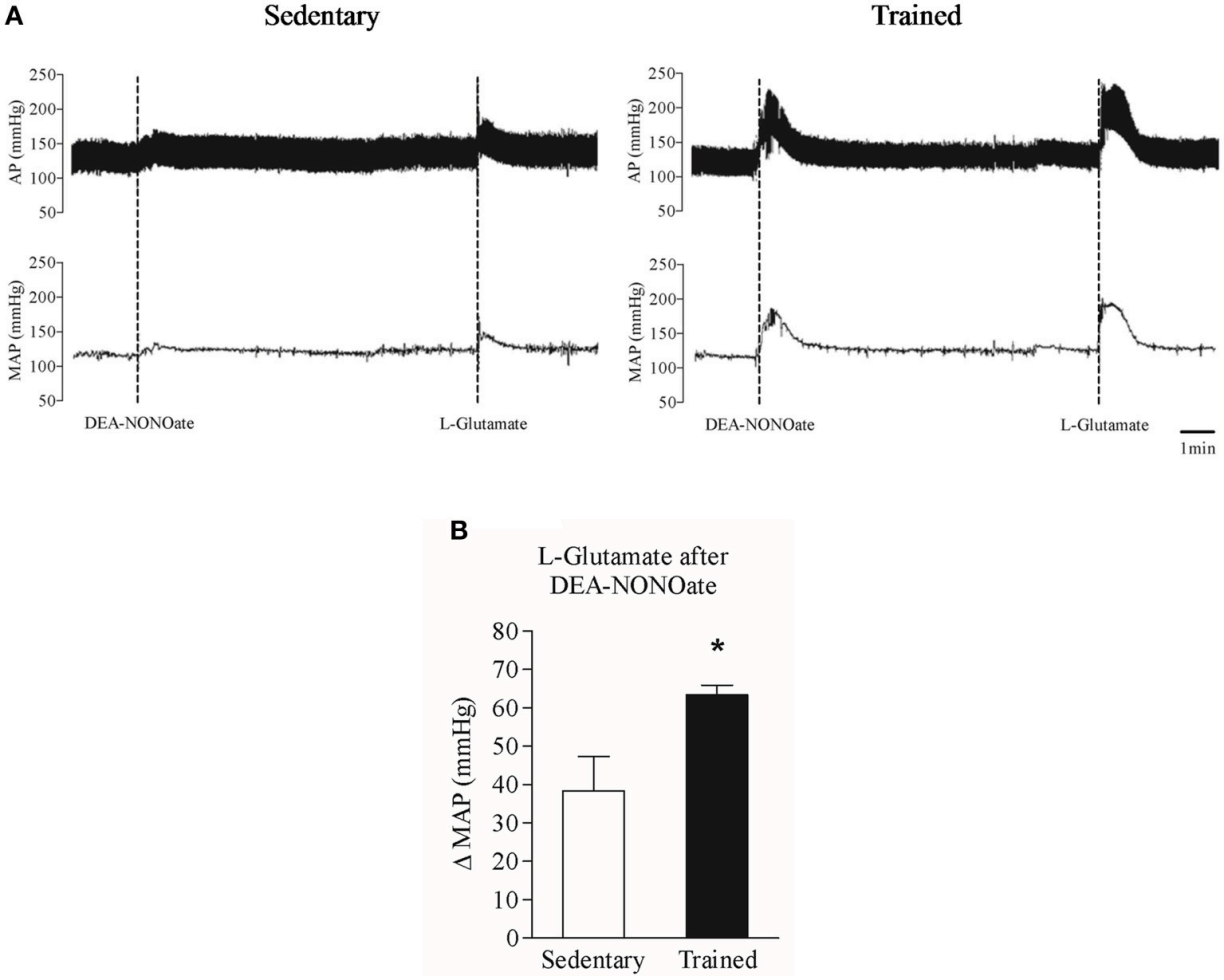
**Mean Arterial Pressure (MAP) responses (ΔMAP) to unilateral L-glutamate microinjection in the RVLM of sedentary and trained rats after pretreatment of RVLM with DEA-NONOate**. **(A)** Typical tracing of one sedentary and one trained rat. **(B)** Mean values of L-glutamate microinjections in sedentary and trained rats after DEA-NONOate. Significance (*p* < 0.05) ^*^ vs. sedentary group.

Considering that NO seems to be decreased in RVLM of trained rats, we performed L-glutamate microinjection in TR and Sed rats after treatment with DeaNonoate (Figure 5A). After the NO-donor, L-glutamate pressor responses were increased in trained rats (Sed: 45.29 ± 5.76 vs. Tr: 65.03 ± 5.24, *P* > 0.05, Figure 5B).

## Discussion

First of all, since several studies in the literature described increased baroreflex sensitivity after aerobic training (Brum et al., 2000; Medeiros et al., 2004; Ceroni et al., 2009; Cavalleri et al., 2011; Masson et al., 2014), we also investigated the ability of swimming training to alter baroreceptor reflex control of HR. We observed that 4 weeks of swimming training markedly improved baroreflex gain and increased the operational range of the reflex. Training-induced improvement of baroreflex sensitivity by providing a better ability to correct instantaneous pressure oscillations reduces pressure variability and consequent capillary lesions thus improving cardiovascular homeostasis. This study also confirmed previous observations in the literature that swimming training did not change pressure levels of normotensive rats, but induced resting bradycardia (Medeiros et al., 2004; Mehanna et al., 2007; Mastelari et al., 2011; Sant'Ana et al., 2011).

In the present study, some new observations were obtained with the swimming training protocol of 4 weeks: (1) similar to other training protocols, swimming training was also able to improve the baroreceptor reflex control of HR; (2) the lower training-induced RVLM NO content significantly contributes to the lower pressor response to glutamate since the difference between trained and sedentary rats disappear after the local administration of NO scavenger or NOS inhibitors; (3) reduced NO availability in trained rats may decrease RVLM glutamatergic activity, thus reducing glutamate-induced sympathoexcitation; (4) both isoforms are able to release NO in the RVLM, nNOS and eNOS seems to be involved in the reduced NO content during RVLM glutamate administration. (5) After adding a NO-donor, the attenuated pressor response to L-glutamate in Tr group was restored. Together these data indicate that swimming training, although not changing basal pressor levels, is able to refrain glutamate-stimulated pressure increases by reducing RVLM NO modulation of sympathetic activity.

In trained rats, the lower pressor response to glutamate administration in the RVLM confirmed previous data from our laboratory (Martins-Pinge et al., 2005). In addition, the present results by comparing in the same rats the pressure responsiveness to glutamate before and after the blockade of NO availability (by either NO scavenger and blockade of NOS isoforms), showed that the smaller pressor response exhibited by trained rats was due to a low NO content in the RVLM. Indeed the measurement of nitrite content within this area confirmed the reduced NO availability after swimming training. On the other hand, pressor responsiveness was not changed in sedentary rats submitted to the same RVLM treatments. Since the difference in the pressor response was only observed in trained rats before treatments, was not present after NO withdrawal in the trained group and was not significantly affect by NO removal in sedentary rats, we suggest that training abrogated pressor responsiveness to glutamate by decreasing NO release and its excitatory effects on glutamatergic neurons. We also confirm this hypothesis when after adding a NO-donor, the increase in MAP by L-glutamate was restored.

Regarding the effects of NO in the RVLM, there are controversies in the literature. Some investigators reported blood pressure increases after administration of L-arginine and NO donors (Hirooka et al., 1996; Martins-Pinge et al., 1999), while others observed a significant decrease (Shapoval et al., 1991; Zanzinger et al., 1995; Tseng et al., 1996; Kagiyama et al., 1997). It is possible that these contradictory effects are grounded in different concentrations used by researchers: while high NO doses in the RVLM lead to decreases, lower doses produce arterial pressure increases (Morimoto et al., 2000). It is important to note that pharmacological studies in the central nervous system of different species showed that NO may interact with both the glutamatergic excitatory and GABAergic inhibitory neurons thus being able to cause neuronal excitation or neuronal inhibition (Tseng et al., 1996; Chen et al., 2001; Ishide et al., 2003; Martins-Pinge et al., 2013). However, considering the predominance of sympathetic premotor neurons in the RVLM that are glutamatergic (Dampney, 1994; Mischel et al., 2015), and the observations that blockade of endogenous NO release by NO scavengers in the RLVM was accompanied by hypotension and bradycardia (Chan et al., 2001) and reduced pressor response (present set of data) we may suggest that NO within the RVLM modulates preferentially the sympathoexcitation mediated by glutamatergic neurons. In addition, RVLM blockade of NOS isoforms by both Nw-Propyl-L-Arginine and L-NAME was accompanied by smaller pressor response to locally administered glutamate. These data together with previous studies showing MAP, HR, and renal sympathetic nerve activity reductions after NOS blockade in the RVLM (Hirooka et al., 1996; Chan et al., 2003; Martins-Pinge et al., 2007) indicate an excitatory effect of locally released NO that is blunted by swimming training.

Both isoforms are able to synthesize NO when properly stimulated by the increased neuronal activity (nNOS) or by the augmented shear stress (eNOS). Our data showed that trained rats exhibited a huge increase in eNOS protein expression and a possible compensatory downregulation of eNOS gene expression due to the increased capillary profile observed after training (Dunn et al., 2012; Huang et al., 2013). Notice that RVLM glutamate injection was made when trained and sedentary rats are resting in their home cages, therefore not exhibiting a hyperkinetic circulation to stimulate eNOS via increased shear stress. On the other hand the slight reduction in nNOS expression (gene and protein) observed in the RVLM of trained rats may account for the reduced NO availability upon neuronal activation by glutamate administration. This does not preclude additional activation of eNOS and endothelial NO production during acute bouts of exercise. Indeed Ishide et al. (2003, 2005) showed that both, nNOS and eNOS are involved in RVLM NO synthesis during muscle contractions. Importantly, our data showed that 4 weeks of swimming training caused a marked reduction of NO availability in the RVLM, as measured by nitrite concentration. The ability of swimming training to reduce both the increased NOS expression and the elevated NO synthesis observed in hypertensive rats was recently demonstrated in the RVLM of 2K-1C trained rats (Sousa et al., 2015).

In summary, our data indicate that swimming training decreases RVLM NO availability, therefore reducing glutamatergic activity in sympathetic premotor neurons and the stimulated pressor response. The present set of data demonstrates an important modulatory role of RVLM glutamatergic neurons by locally released NO in exercise trained subjects.

## Author note

The present study evaluated glutamate and nitric oxide interactions in the RVLM of normotensive rats previously submitted to swimming training. The functional studies were performed in conscious rats, avoiding anesthesia influence on neural function, focusing on glutamate effects in RVLM and the contributions of eNOS and nNOS isoforms and its cardiovascular control. We observed that exercise training decreased nitric oxide production in RVLM, collaborating to decrease sympathetic activity in trained subjects. Also, no studies have been evaluating those aspects in conscious rats.

## Author contributions

Conception and design: HR, LM, MM. Acquisition, analysis and interpretation of data: HR, GM, BB, NZ,. Analyzed the data: HR, GM, BB, NZ, PP, LM, MM. Materials and reagents: PP, LM, MM. Drafting or revising and final approval: HR, GM, BB, NZ, PP, LM, MM.

### Conflict of interest statement

The authors declare that the research was conducted in the absence of any commercial or financial relationships that could be construed as a potential conflict of interest.
